# Image analysis using smartphones: relationship between leaf color and fresh weight of lettuce under different nutritional treatments

**DOI:** 10.3389/fpls.2025.1589825

**Published:** 2025-05-05

**Authors:** Hye Su Won, Eunji Lee, Seeun Lee, Ji-Hyeon Nam, Jiwon Jung, Yuna Cho, Thomas Evert, Noah Kan, Steven Kim, Dong Sub Kim

**Affiliations:** ^1^ Department of Horticulture, Kongju National University, Yesan, Republic of Korea; ^2^ Department of Biological Science, Kongju National University, Gongju, Republic of Korea; ^3^ Department of Mathematics and Statistics, California State University, Monterey Bay, Seaside, CA, United States

**Keywords:** normalized intensity, dark green proportion, RGB, Bland-Altman analysis, green and red lettuces

## Abstract

Image analysis can be useful for assessing crop health and predicting yield. Instead of expensive equipment, smartphones are considered an accessible and low-cost alternative. The objectives of this study were to evaluate whether fresh weight in green and red lettuce could be predicted by leaf color (intensity of green color measured by RGB) under different fertilizer treatments using RGB imaging from two widely used smartphone models (Samsung Galaxy and Apple iPhone). The two smartphones showed similar longitudinal patterns of RGB data (the intensity and dark green proportion), but the absolute difference in the RGB data was significantly different. Therefore, the averaged results were used for the analyses. Color intensity and dark green proportion were associated with the fresh lettuce weight (p = 0.005, 0.003, 0.014 and p < 0.001, respectively). This study suggests that farmers and practitioners can use these economic devices as a non-destructive method to diagnose and monitor the nutritional status and predict lettuce yield.

## 1 Introduction

The nutritional status of crops is a critical factor in determining their productivity and quality. In particular, nitrogen is an essential element for crop growth, and is a growth-limiting factor as the major element in amino acids, nucleic acids, and chlorophylls (Maathuis, 2009). Thus, nitrogen deficiency induces reduction of crop growth and productivity, chloroplast disintegration, and even plant death (Vos et al., 2005). Conversely, excessive nitrogen leads to abnormal vegetative growth, low flower number, and increased susceptibility to plant pathogens (Albornoz et al., 2016). Moreover, nitrogen lost from the soil causes severe blooms of aquatic algae and macrophytes associated with eutrophication in lakes, streams, rivers, and oceans (Carpenter et al., 1998; Schindler et al., 2008). Nitrogen deficiency often results in pale green or yellowing leaves due to reduced chlorophyll content, whereas excessive nitrogen can lead to overly dark green leaves with excessive vegetative growth (McCauley et al., 2009).

Micronutrients play a crucial role in plant growth and quality. Microelement deficiency, another commonly observed nutritional disorder, significantly impacts plant growth, yield, and quality (Nadeem and Farooq, 2019). Excessive iron, zinc, and manganese reduces photosynthetic efficiency and impedes nutrient absorption, ultimately leading to reduction of plant growth and productivity (Wairich et al., 2024; Mousavi, 2011; Millaleo et al., 2010). Like deficient and excessive nitrogen, deficient and excessive micronutrients can be visually diagnosed through leaf color, but it is challenging to quantify leaf color for objective assessment.

In agriculture, data collection from bioassays is time consuming and laborious. Furthermore, evaluation of crop nutritional status is not reliable or very expensive for high reliability. For example, chlorophyll meters (e.g., SPAD meters) have been used for non-destructive measurement (Balasubramanian et al., 1998), but these devices only assess a small portion of the leaf, making it difficult to represent the overall condition of the crop. On the other hand, hyperspectral, multispectral, and thermal imaging technologies allow for non-destructive and accurate analysis of plant nutritional status, including water stress and nutrient deficiencies. However, these technologies require expert knowledge and the use of expensive, complex equipment for data collection and analysis, which limits their accessibility for farmers (Sankaran et al., 2015; Mahlein, 2016). As a result, there is increasing interest in cost-effective alternatives, such as RGB imaging, which offer a more accessible approach to plant monitoring.

To overcome these practical limitations, RGB imaging offers a more accessible and cost-effective alternative. Through advancements in smartphone camera technology, the accessibility of RGB image acquisition has significantly improved. RGB imaging has limitations in directly detecting and quantifying physiological responses occurring within plant tissues. However, nutrient excess or deficiency often manifests as visible color changes on the surface of the plant (Uchida, 2000). Therefore, RGB imaging can be used to detect such color changes and potentially indirectly predict nutrient disorders in crops. The RGB color model can be utilized to quantify crop growth status, and free or low-cost image analysis software has made RGB analysis more accessible and convenient. For instance, Ibrahim et al. (2021) developed a smartphone-based image acquisition method to estimate chlorophyll content in lettuce leaves using controlled lighting conditions with an SMD LED setup. By extracting RGB indices and calculating vegetation indices (e.g., VI, GDR), they reported a strong correlation between the image-based indices and SPAD-measured chlorophyll content. Kim et al. (2022) found a relationship between RGB ratios and strawberry yield, and Petrozza et al. (2014) monitored physiological responses and biomass of tomato plants using RGB imaging. Similarly, Elsayed et al. (2018) demonstrated that green pixel proportions derived from digital analysis are strongly associated with nitrogen uptake and biomass in wheat. Therefore, RGB imaging has the potential to serve as an accessible indicator for evaluating crop nutritional status. However, further validation is needed to confirm its reliability.

According to Fontes et al. (1997), nitrogen application influences crop productivity by affecting physiological responses. In wheat, RGB-based indices such as G/R, G/B, and (G–R)/(R+G+B) from canopy images have shown strong correlations with both leaf nitrogen content and yield (Qi et al., 2021). Based on these findings, this study applies a similar approach to lettuce by analyzing two image-derived indices: (1) normalized intensity, defined as I = (R+G+B)/3, and (2) dark green proportion, calculated as the ratio of the pixels occupied by a predefined dark color range (RGB intensity between 0 and 85) to the total pixels in a segmented leaf area. Building on these methods, this study investigates whether color-based indicators derived from RGB images can be used to estimate fresh weight in lettuce under different fertilizer treatments.

This study aims to evaluate whether fresh weight in lettuce could be predicted by leaf color (intensity of green color measured by RGB) under different fertilizer treatments using RGB imaging from two widely used smartphone models (Samsung Galaxy and Apple iPhone). By analyzing the relationship between leaf color and plant biomass, this study explores the feasibility of smartphone-based imaging as a cost-effective and non-destructive tool for assessing crop growth.

## 2 Materials and methods

### 2.1 Plant materials and cultivation conditions

**Figure 1:**
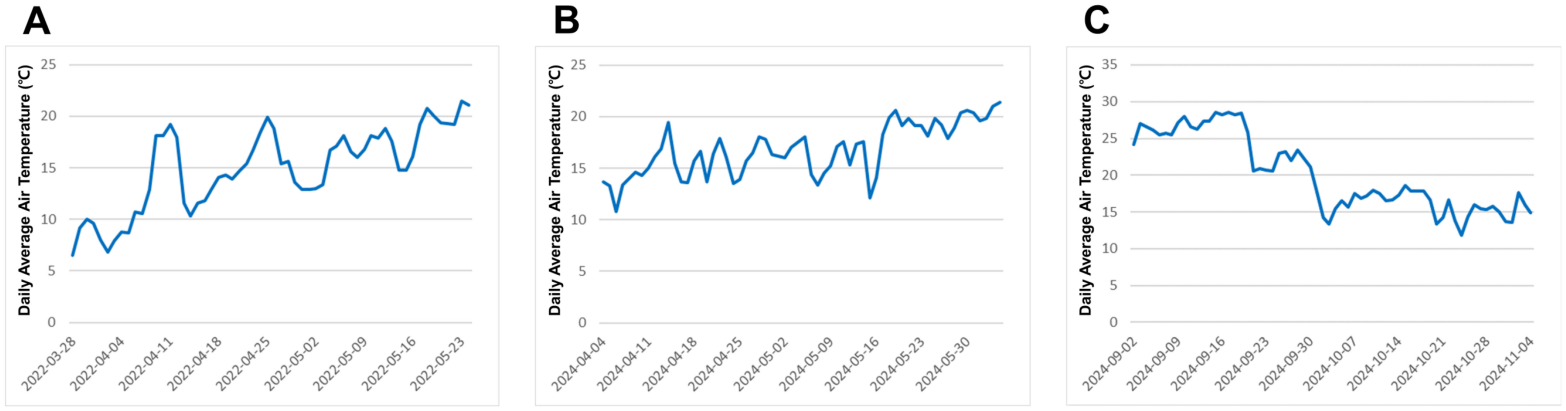
Daily average air temperature during the lettuce cultivation in 2022 trial and 2024 trial **(A)** from March 28 to May 24, 2022, **(B)** from April 4 to June 4, 2024, **(C)** from September 2 to November 4, 2024.

The two lettuce cultivars ‘Cheongchima’ (a green-leaf lettuce) and ‘Jeokchima’ (a red-leaf lettuce) were grown in a polyethylene-covered greenhouse in Yesan Campus of Kongju National University, South Korea (36°40’2.4”N 126°51’49.5”E). Each lettuce type was tested twice. The first green lettuce experiment was carried out in the spring of 2022, and the second green lettuce experiment was done in the spring of 2024. The first red lettuce experiment was conducted in the spring of 2024 (at the same time of the second green lettuce experiment), and the second red lettuce experiment was done in the fall of 2024. The daily average air temperature ranged from 6.5 to 21.5°C in the spring of 2022; from 10.8 to 21.4°C in the spring of 2024; and from 11.9 and 28.6°C in the fall of 2024 (**Figure 1**).

In the spring of 2022, the first green lettuce experiment was conducted from March 28 to May 24, 2022. Five seeds of ‘Cheongchima’ (World Seed Co., South Korea) were sown in four plastic pots filled with commercial soil (Hungnongseed. Co., South Korea), and covered with vermiculites (GFC. Co., South Korea). The top diameter of the pot was 13 cm, and the height was 13.5 cm. The lettuces were irrigated with 120 mL of water at 8 A.M. every three days. Three weeks after sowing, the first main leaf was fully developed and four seedlings were thinned. Three days after thinning, the irrigation was increased to 150 mL every three days. With regard to all fertilizers, the rate recommended by the manufacturer was applied for the selected pot size. For a precise treatment, fertilizers of different particle sizes were blended into a nutrient solution. For example, when 0.34 g nitrogen fertilizer was to be applied per pot, a 20-fold concentrate was diluted and applied to the pots with irrigation water. Similarly, 0.01 g iron fertilizer and 0.66 g fertilizer for leafy vegetables were applied per pot. After the second fertilization, as the size of lettuce and air temperature increased, the irrigation was increased to 300 mL daily. Three fertilizers were used to evaluate the effect of: a nitrogen fertilizer (46% nitrogen) (TaegheungF&G. Co., South Korea), a fertilizer for leafy vegetables (12% nitrogen, 5% water-soluble phosphoric acid, 7% water-soluble potassium, 3% magnesium oxide, and 0.3% citrate-soluble boron) (TaegheungF&G. Co., South Korea) and a microelement fertilizer (5.5% water-soluble iron, 3.5% water-soluble zinc, 0.3% water-soluble manganese, and 0.005% water-soluble molybdenum) (Daeyu Co., South Korea). The fertilizers were applied twice, once after the first main leaf developed and then after three weeks.

In the spring of 2024, the green lettuce (‘Cheongchima’, World Seed Co., South Korea) was grown from April 4 to May 30 in 2024, and the red lettuce (‘Jeokchima’, Kwonnong., South Korea) was grown from April 4 to June 4, 2024. The eight lettuces per treatment were sub-irrigated three times a week for three hours from 9 A.M. to 12 P.M. Three weeks after sowing, the third main leaf was developed and the lettuces were transplanted into pots. The first fertilization was performed one week after transplanting, and the second fertilization was two weeks after the first fertilization. However, since the red lettuces grew slower, its first fertilization was done five days later than the green lettuces. The ingredient, amount, and method of fertilizer and size of pot were the same as in the 2022 experiment.

In the fall of 2024, the second red lettuce experiment was conducted. The red lettuce was grown from September 2 to November 4, 2024. The six lettuces per the treatment were sub-irrigated three times a week for three hours from 9 A.M. to 12 P.M. Four weeks after sowing, the third main leaf was developed and the lettuces were transplanted into pots. As in the 2024 spring experiment, the first fertilization was performed one week after transplanting, and the second fertilization was two weeks after the first fertilization. The ingredient, amount, and method of fertilizer and size of pot were the same as in the 2022 experiment.

### 2.2 Acquirement of growth data

The fresh weight was measured and the number of leaves were counted on harvest day. The fresh weight was measured from the above-ground part excluding roots. The leaves measuring less than 2 cm on the longest side were excluded from the leaf count.

### 2.3 Acquirement of image data

In the spring 2022, the green lettuces were photographed on the last day of the experiment. There were four lettuces per treatment. All photos of these plants were taken on clear days, which might have resulted in slight differences from the actual harvest days. The image acquisition dates differed by up to one week between the plants. We monitored the plants by collecting image data twice a week, and we observed consistent patterns of RGB values after a certain point during the growth period. There would be inherent variations in the image data between days, and even within days, but given the consistent patterns observed near the last day of the experiment, we believe that the image data taken within one week represented the leaf colors and that the different time points did not have significant impact on the correlation between RGB values and fresh weight.

The photos of each lettuce were taken using two smartphone models, a Samsung Galaxy A50 (referred to as Model 1) and an Apple iPhone 13 mini (Model 2). Model 1 was equipped with a 25 MP wide-angle camera, and Model 2 had a 12 MP wide-angle camera. The images captured by Models 1 and 2 had resolutions of 3024 × 3024 pixels and 1440 × 1440 pixels, respectively. Each lettuce was photographed separately, and the cameras were positioned vertically above the plant to capture the canopy. The images were captured from a height of 0.5 m under natural light conditions using the smartphones’ automatic shooting mode. The photos were converted from JPEG to GIF format for image analysis (Kim et al., 2022). To remove irrelevant parts in each photo, green vegetation and other parts (e.g., soil and plastic pots) in the GIF files were separated out using image segmentation (Kim et al., 2021). The separated GIF files were uploaded to the image analysis program freely available at http://mkwak.org/imgarea.

In the spring of 2024, green lettuces were photographed two days before and red lettuces seven days before the experiment ended. A Samsung Galaxy Note 10 (Model 3) and an Apple iPhone 8 (Model 4) were used to take photos. Both Models 3 and 4 were equipped with 12 MP wide-angle cameras, and all images were captured at a resolution of 4032 × 4032 pixels. In the second green lettuce experiment and the first red lettuce experiment, four lettuces per treatment on black background were photographed at a time with a color chart (Spyder Checker 24). The camera was positioned vertically above the plant to capture the canopy, and the images were captured at a height of 1 m. As in the spring 2022 experiment, the images were captured under natural light conditions using the smartphones’ automatic shooting mode. The method of editing and analyzing photos was the same as in the 2022 experiment.

In the fall of 2024, the red lettuces were photographed three days before the experiment ended.) The same smartphone models, Models 3 and 4, were used to take photos, and six lettuces per treatment were photographed at a time against a black background, along with a color chart (Spyder Checker 24). The experimental conditions (the shooting distance, angle, lighting conditions, and image resolution) were consistent with those in the spring of 2024. The method for editing and analyzing the photos was the same as in the 2022 experiment. Given the inherent variability of smartphone cameras and experimental conditions across the experiments, the standardization of the image capturing process is critical, and the discrepancy is the RGB values were analyzed and the average of two smartphones’ results was used in statistical analysis as described in the subsequent section.

### 2.4 Statistical analysis

The image analysis program receives a GIF image file and outputs uniquely observed RGB codes and associated pixels in the input image. The pixels of green colors, for which the value of G was greater than the values of R and B, were included in the analysis, and the two response variables were derived from the raw data. The first variable was the intensity, defined as I = (R + G + B)/3 after normalizing R, G, and B (Zhi et al., 2020). When the R, G, and B values are normalized between zero and one, I = 0 represents black, and I = 1 represents white.

The second variable was the proportion of dark green color. The values of R, G, and B lie between 0 and 255, and the RGB codes (0, 0, 0) and (255, 255, 255) represent black and white, respectively. The value of G was divided into three levels: 0 to 85 for dark green, 86 to 170 for medium, and 171 to 255 for light green. **Table 1** presents four commonly observed dark green, medium green, and light green colors and the corresponding RGB codes, and the proportion of dark green color is defined as the proportion of pixels occupied by the four dark green colors: (0, 43, 0), (0, 85, 0), (51, 85, 0), and (51, 85, 51). This proportion is hereafter referred to as the dark green proportion.

**Table 1:** Examples of dark, medium, and light green colors commonly observed in the image data.

Dark		Medium		Light	
Color	RGB	Color	RGB	Color	RGB
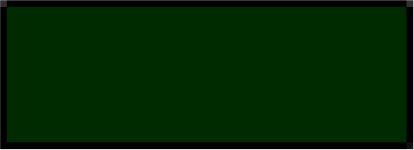	(0, 43, 0)	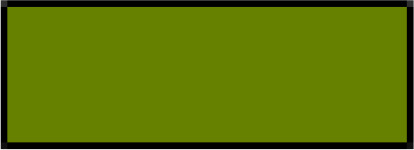	(102, 128, 0)	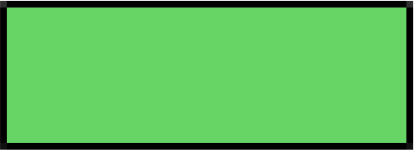	(102, 213,102)
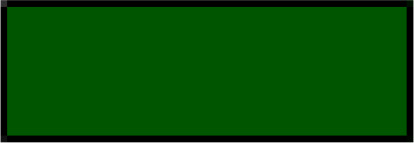	(0, 85, 0)	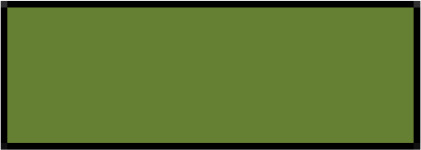	(102, 128, 51)	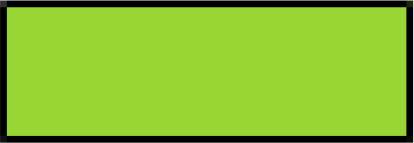	(153, 213,51)
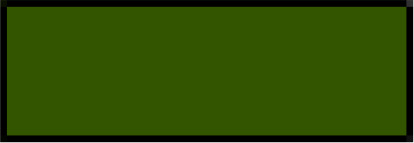	(51, 85, 0)	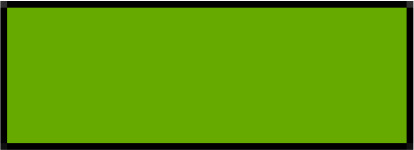	(102, 170, 0)	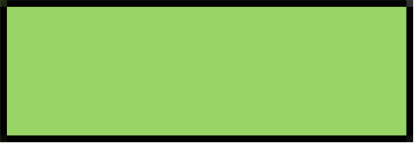	(153, 213, 102)
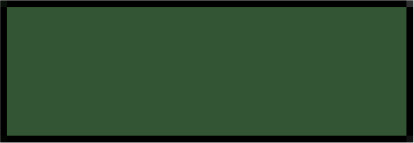	(51, 85, 51)	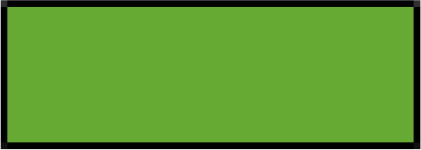	(102, 170, 51)	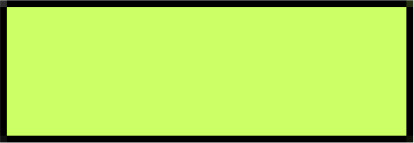	(204, 255, 102)

The values of intensity and dark green proportion observed from the same lettuce leaf were not identical between Models 1 and 2 and between Models 3 and 4. We evaluated the degree of agreement between the two smartphone models using Bland-Altman analysis (Bland and Altman, 1986), which is a visual representation and statistical inference used to assess the agreement between two measurement devices. We let x and y be observed values of the same lettuce leaf when the photo was taken by two different smartphone models. Given n paired observations of (x, y), we let m be the calculated average of the difference, d = y – x, and let s be the standard deviation of d. the be the difference. The 95% limits of agreement (LOA) were calculated by


m±1.96×s

and the 95% confidence interval (CI) for the expected difference was calculated by


$$\text{m}\pm 1.96\times \text{s}/{\text{n}}^{1/2}.$$

We note that the 95% LOA and 95% CI have different roles as follows. The 95% LOA is devised to predict random differences to be observed if the experiment is repeated, and the 95% CI is devised to estimate the expected (average) difference. If a calculated 95% CI is entirely positive or negative (i.e., zero is not in the interval), it is statistical evidence that one model tends to underestimate or overestimate the color parameter values of the same target when compared to the other model. If a calculated 95% LOA is wide, the difference between the two measures, x and y, is large, hence it indicates unreliable measures.

Analysis of variance was used to compare the intensity and dark green proportion between the four treatment groups. The mixed-effects model was used to test for the relationships between fresh weight and intensity and between fresh weight and dark green proportion within the treatment groups. R version 4.3.0 was used for the statistical analyses (R Core Team, 2023).

## 3 Results

### 3.1 Bland-Altman analysis

**Figure 2:**
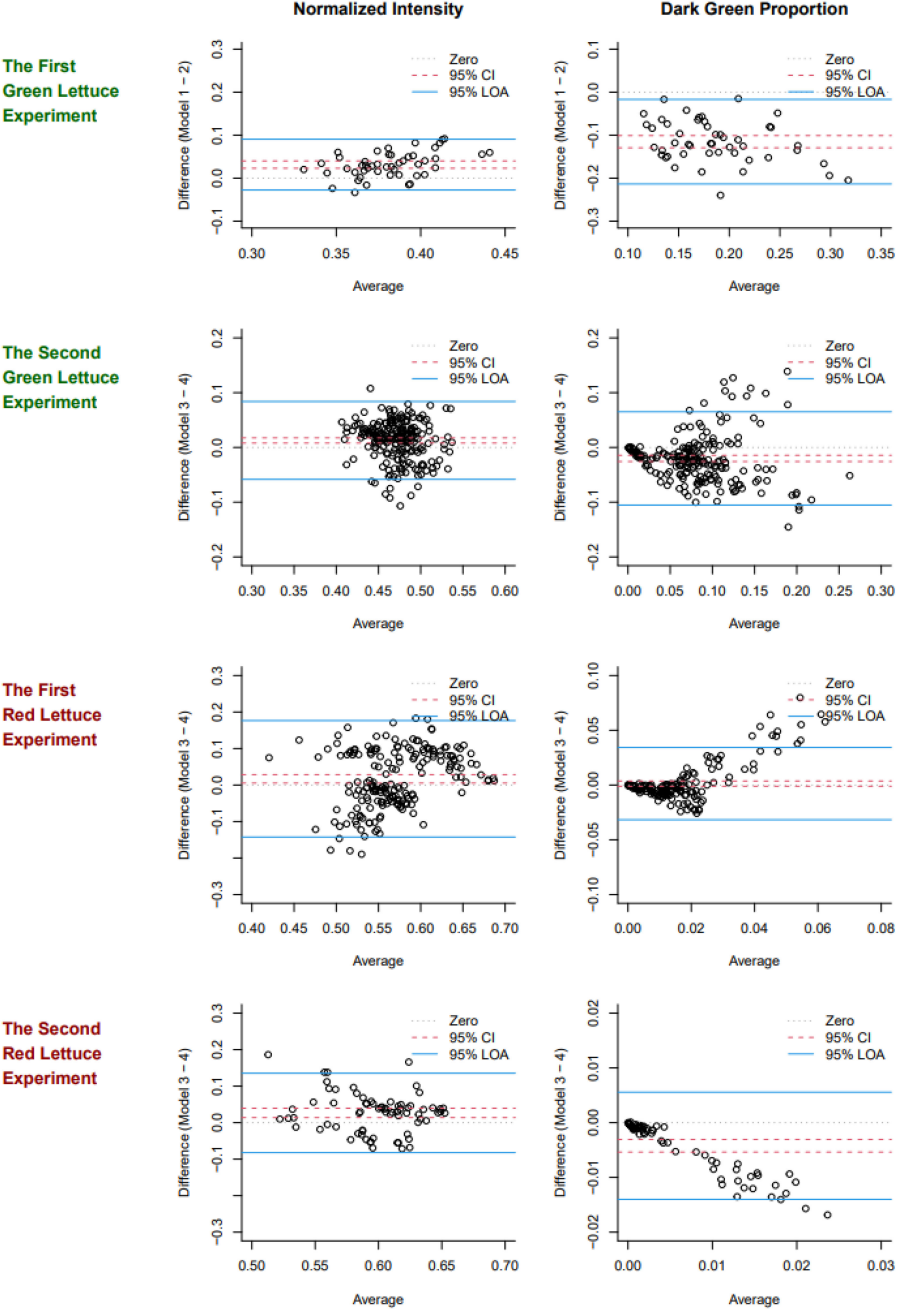
Bland-Altman plots of normalized intensity values (the first column) and dark green proportions (the second column) observed in the four experiments (each row). The 95% confidence intervals (CIs) are marked by dotted lines, the 95% limits of agreement (LOAs) are marked by solid lines, and another dotted line is used to mark the zero.

**Figure 2** shows the Bland-Altman plot used to assess the degree of agreement between two measurement devices across four experiments: the first and second green lettuce experiments, as well as the first and second red lettuce experiments. Models 1 and 2 are compared for the first green lettuce experiment (Spring 2022), and Models 3 and 4 are compared for the remaining experiments (Spring 2024 and Fall 2024).

In the first green lettuce experiment in spring 2022, for normalized intensity, the resulting 95% CI and LOA were (0.02, 0.04) and (-0.03, 0.09), respectively. That is, Model 2 (Apple iPhone 13 Mini) tends to have a higher estimate of normalized intensity than Model 1 (Samsung Galaxy A50), on average, and the differences between the two models range between -0.03 and 0.09 95% of the time. The 95% CI and LOA for the dark green proportion were (-0.13, -0.10) and (-0.21, -0.02), respectively. This result suggests that two measurement devices have a systematic disagreement, on average, and averaging two results would be recommended for subsequent analyses. **Table 2** summarizes the results of Bland-Altman analysis for the normalized intensity and dark green proportion in each experiment.

**Table 2:** The results of Bland-Altman analysis (95% CIs and 95% LOAs) for the normalized intensity and dark green proportion in each experiment.

Experiment		Normalized intensity		Dark green proportion	
	Number of photos compared (n)	95% CI for mean difference	95% LOA for difference	95% CI for mean difference	95% LOA for difference
Green lettuce (Spring 2022) Models 1 vs. 2	48	(0.02, 0.04)	(-0.03, 0.09)	(-0.13, -0.10)	(-0.21, -0.02)
Green lettuce (Spring 2024) Models 3 vs. 4	224	(0.01, 0.02)	(-0.06, 0.08)	(-0.03, -0.01)	(-0.11, 0.07)
Red lettuce (Spring 2024) Models 3 vs. 4	192	(0.01, 0.03)	(-0.14, 0.18)	(-0.001, 0.003)	(-0.03, 0.03)
Red lettuce (Fall 2024) Models 3 vs. 4	72	(0.01, 0.04)	(-0.08, 0.14)	(-0.01, -0.003)	(-0.01, 0.01)

Similar trends were observed between Models 3 and 4. Model 4 (Apple iPhone 4) resulted in a higher value of normalized intensity and a lower value of the dark green proportion than Model 3 (Samsung Galaxy Note 10), on average, in the experiments in spring 2024 and fall 2024. The sample size (n) affected the precision of the 95% CIs reported in **Table 2**, and the systematic disagreement was observed even when n = 48 in spring 2022. We observed that images taken by iPhones appeared to be more saturated in color than images taken by Galaxy phones, and this difference could contribute to the consistently skewed 95% CIs. This result suggests that calibration and adjustment between the two models would have provided more robust results in these experiments and would increase statistical power for the analyses reported in the subsequent sections.

According to the 95% LOAs, the two devices disagreed more when the normalized intensity was measured for red lettuce than for green lettuce, and they disagreed more when the dark green proportion was measured for green lettuce than for red lettuce. Given that the true value of each color parameter is unknown and which model’s result is closer to the truth, we used the average of two values for each target to increase the robustness of the results. Even though two devices disagreed in terms of absolute values, their patterns (recognizing high or low intensity and dark green proportion) were consistent enough to obtain some statistical significance in our experiments (see Sections 3.2 and 3.3). Nevertheless, more reliable measurement is desired for future experiments to powerfully detect and precisely estimate the treatment effects and any relationship with growth responses, and practical suggestions for calibration or adjustment prior to future experiments is discussed in Section 4.

### 3.2 Growth responses after fertilizer application

**Figure 3:**
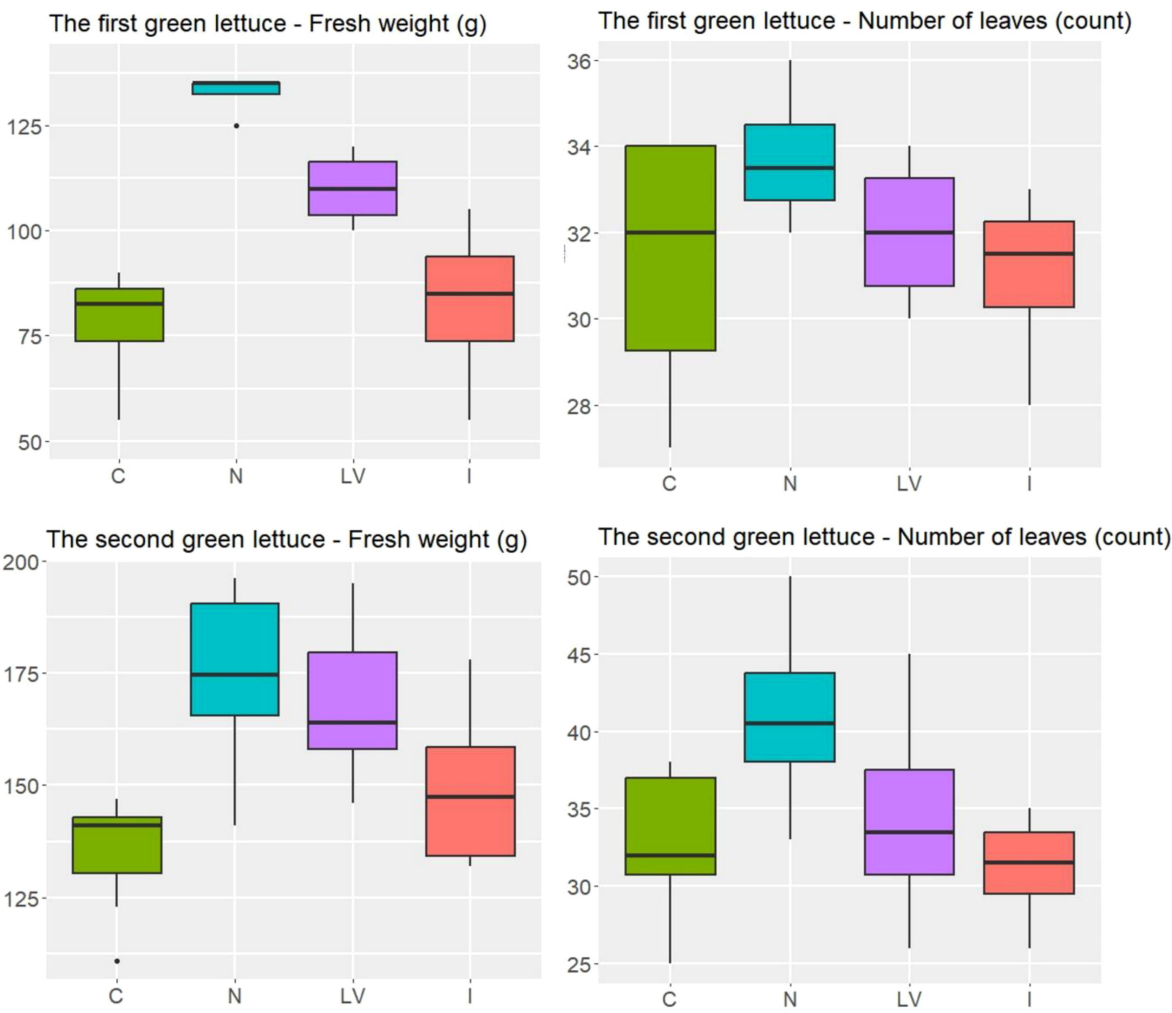
In the green lettuce experiment, the number of leaves and weight (g) in the four treatment groups, C: untreated treatment, LV: fertilizer for leaf vegetables treatment, N: nitrogen fertilizer treatment, I: microelement fertilizer treatment.

The ANOVA was used to compare the four treatments: control (C), nitrogen fertilizer (N), leaf vegetable fertilizer (LV), and microelement fertilizer (I). For the first green lettuce experiment in spring 2022, the expected number of leaves were not significantly different between the four treatments (p = 0.388), and the expected fresh weight was (p < 0.001). In particular, the N and LV treatments resulted in higher averages of fresh weight than the C treatment (p < 0.001 and p = 0.007, respectively). For the second green lettuce experiment in spring 2024, we observed similar patterns for both number of leaves and fresh weight between the four treatments (**Figure 3**). In particular, the N treatment resulted in higher averages of fresh weight than the LV, I, and C treatments (p = 0.004, p < 0.001, and p < 0.001, respectively). In summary, in both green lettuce experiments, the treatment effects were significant for the fresh weight, but not for the number of leaves. In particular, the N treatment appeared to be the most beneficial for increasing the fresh weight among the four treatments compared in the two experiments.

**Figure 4:**
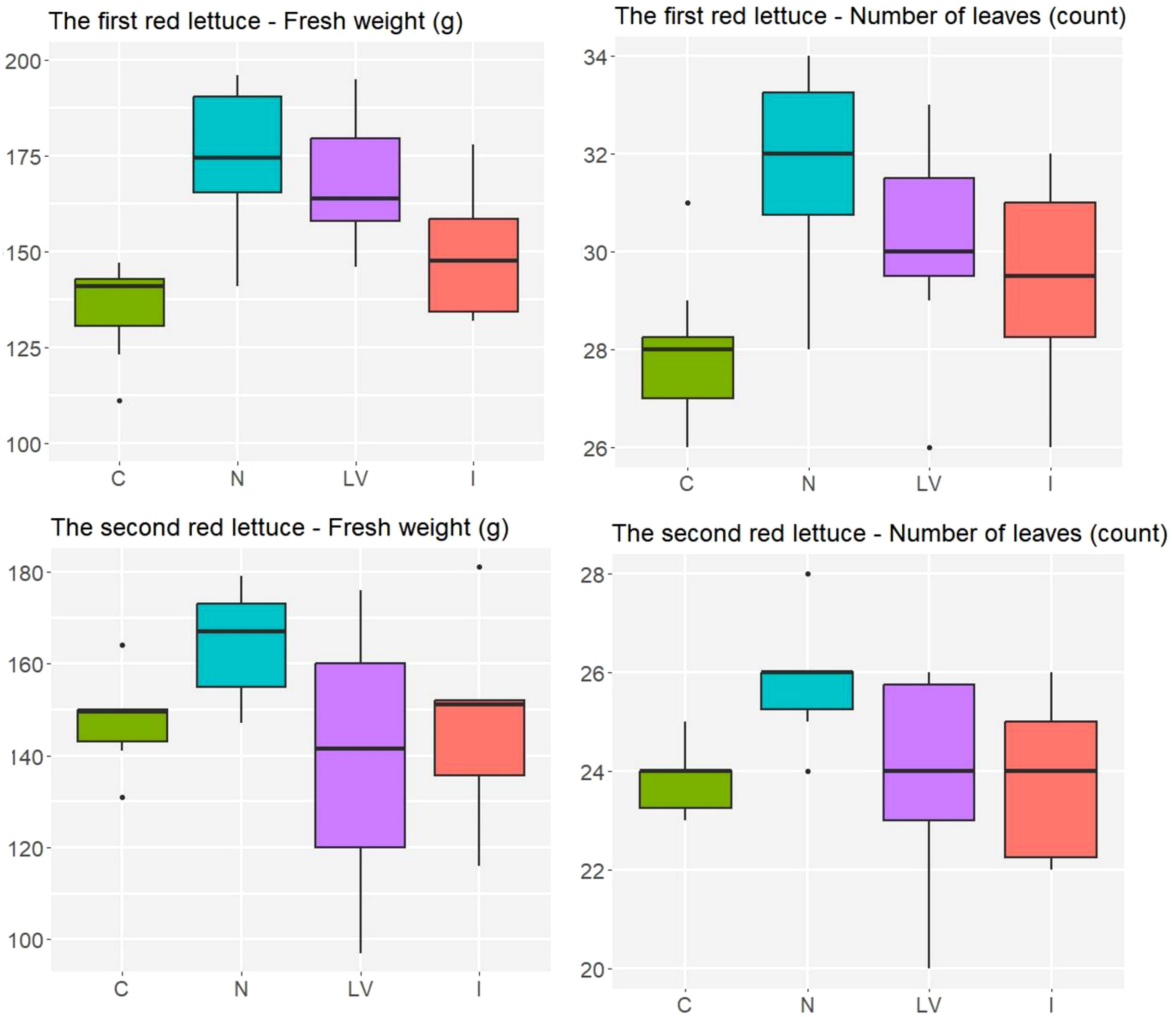
In red lettuce experiment, number of leaves and fresh weight (g) in the four treatment groups, C: untreated treatment, LV: fertilizer for leaf vegetables treatment, N: nitrogen fertilizer treatment, I: microelement fertilizer treatment.

For the first experiment of red lettuce, the N treatment yielded higher fresh weight, on average, than the I and C treatments did (p = 0.019 and p < 0.001, respectively), and the N and LV yielded similar fresh weight, on average (p = 0.884). For the second experiment of red lettuce, due to the high variability in the LV treatment, the ANOVA showed weak evidence for the difference in treatment effects on fresh weight (p = 0.213). The treatment effects on the number of leaves were not statistically significant in both red lettuce experiments as in both green lettuce experiments. Still, the N treatment consistently showed the highest average fresh weight and average number of leaves for the red lettuce (**Figure 4**) as well as for the green lettuce (**Figure 3**). In summary, the N treatment appeared to be the most effective for increasing the growth responses of both green and red lettuce.

### 3.3 Color parameters and after fertilizer application

**Figure 5:**
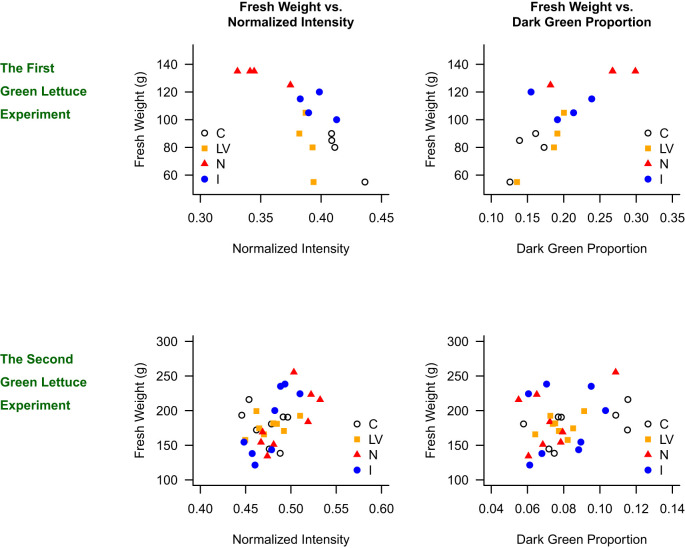
Relationship between fresh weight and normalized intensity, and fresh weight and dark green proportion in the green lettuce experiments.

**Figure 5** presents the scatter plots of fresh weight against normalized intensity for the green lettuce, and the four treatments (C, LV, N, and I) are distinguished by the shapes of data points. In the first green lettuce experiment, it was clearly shown that the C treatment resulted in high intensity value, low dark green proportion, and low fresh weight, whereas the N treatment resulted in low intensity value, high dark green proportion, and high fresh weight. In addition, when we accounted for treatment effects in the mixed-effects model, the expected fresh weight was higher for plants with lower normalized intensity (p = 0.003, R² = 0.518) and those with higher dark green proportion (p = 0.005, R² = 0.356). In the second green lettuce experiment, the scatter plots were not as clear as in the first experiment. The fresh weight and normalized intensity were not significantly correlated (p = 0.2229, R² = 0.009). Although the relationship between fresh weight and dark green proportion was statistically significant, the R-square value was low (p = 0.003, R² = 0.064).

**Figure 6:**
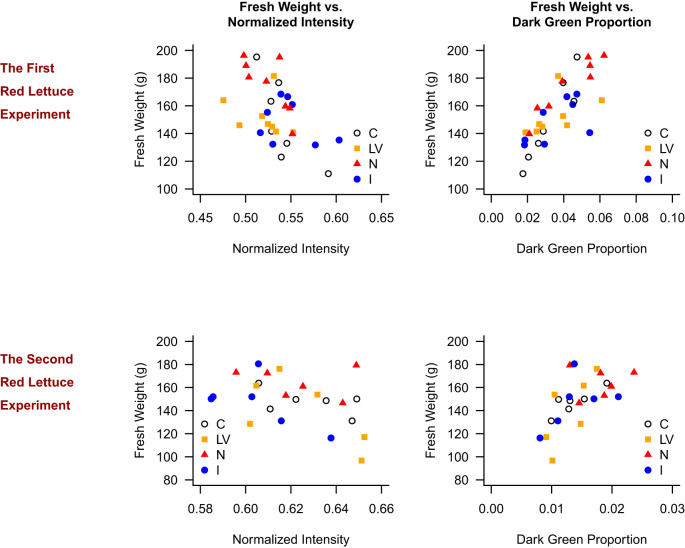
Relationship between fresh weight and normalized intensity, and fresh weight and dark green proportion in the red lettuce experiments.

**Figure 6** presents the scatter plots for the red lettuce. As shown in **Figure 5** for the green lettuce, the C treatment generally resulted in high intensity value, low dark green proportion, and low fresh weight, whereas the N treatment resulted in low intensity value, high dark green proportion, and high fresh weight. This pattern was clearer in the first red lettuce experiment. In the first red lettuce experiment, fresh weight was higher in plants with lower normalized intensity (p = 0.014, R² = 0.121) and higher dark green proportion (p < 0.001, R² = 0.581). The similar patterns were replicated in the second red lettuce experiment that fresh weight was higher in plants with lower normalized intensity (p = 0.009, R² = 0.202) and higher dark green proportion (p < 0.001, R² = 0.375).

In general, it appears that the correlation between the normalized intensity and fresh weight is negative, but it was not consistently shown in the second green lettuce. On the other hand, the observed correlation between the dark green proportion and fresh weight was consistently positive in all four experiments. In conclusion, the N treatment results in the high dark green proportion, on average, and a high dark green proportion is associated with high fresh weight for both green- and red-leaf lettuce. It appears that the dark green proportion is a more reliable indicator of fresh weight than the intensity value.

## 4 Discussion

Eshkabilov et al. (2021) employed hyperspectral imaging to non-destructively estimate the nutrient content (e.g., NO₃⁻, Ca²⁺, K⁺) of hydroponically grown lettuce, achieving high prediction accuracy. Ren et al. (2017) utilized a low-cost multispectral imaging system to remotely detect nutrient deficiency and water stress in lettuce, demonstrating reasonable classification performance using vegetation indices such as BWDRVI. Sandmann et al. (2018) attempted to detect biotic stress in lettuce using thermal imaging, NDVI, and chlorophyll fluorescence. While fluorescence-based indicators showed high accuracy, thermal indices exhibited relatively poor performance, with error rates exceeding 35%. The use of smartphone-acquired RGB images to evaluate lettuce growth provides a practical and accessible solution for field-based monitoring. However, images captured under natural light conditions are exposed to varying lighting environments, which may introduce noise and inconsistencies in RGB results.

Yang et al. (2021) predicted grain moisture content and optimal harvest timing using smartphones and machine learning. To minimize the influence of lighting variability during outdoor image acquisition, they employed a Simple Spectral–Geometric Correction Board (SSCB) and a color calibration chart. Additionally, Lameski et al. (2017) attempted to address this issue under natural lighting conditions by using techniques such as RGB normalization, vegetation indices, and automatic thresholding (Otsu’s method). We expect that with additional calibration, adjustment, standardization, and preprocessing methods, which are limitations of this study, smartphone imaging can yield more accurate results even under outdoor conditions in future studies.

Prior to this study, it was uncertain whether commonly available smartphone cameras could be used for research or practical purposes (Fan et al., 2021). In this regard, we performed the Bland-Altman analysis, and showed that Models 1 and 3 (Samsung Galaxy) consistently showed a higher normalized intensity value, on average, than Models 2 and 4 (Apple iPhone), respectively, which resulted in Models 1 and 3 showing lower dark green proportions than Models 2 and 4, on average, in three of the four experiments (**Figure 2**). We believe that the different results may depend on smartphone manufacturers because Models 1 and 3 and Models 2 and 4 were made by the same manufacturers. Heinonen and Mattila (2021) reported that various smartphone models perceive colors differently. Despite the degree of disagreement, the longitudinal patterns of the intensity and dark green proportions were similar within the devices, and the average values provided more robust results for comparing the treatment effects. As any color measure can substantially vary among camera models, the Bland-Altman analysis and averaged results are recommended before conducting research, and using the averaged result would be more reliable.

We focused on the two color parameters, normalized intensity and dark green proportion. The dark green proportion showed a significant correlation with fresh weight across all four experiments. In contrast, the normalized intensity was significantly correlated with fresh weight in three of the four experiments, and showed weak evidence in the second green lettuce experiment. The normalized intensity had a higher correlation coefficient (R value) than the dark green proportion only in the second green lettuce experiment. In all other experiments, the dark green proportion resulted in a higher R value than the normality intensity. Although the relationship between color variables and fresh weight was statistically significant in most experiments, the low R² values observed in this study suggest that there are other useful color parameters than the normalized intensity and dark green proportion. Ribeiro et al. (2023) confirmed that vegetation indices derived from RGB images, such as GLI, NGRDI, and SI, showed significant correlations with biometric traits of lettuce, including fresh weight. Clemente et al. (2023) demonstrated that vegetation indices such as GLI can reliably estimate anthocyanin, carotenoid, and chlorophyll contents in mini lettuce. Therefore, incorporating various vegetation indices derived from RGB images is expected to enable a more quantitative and precise analysis of the relationship between color information and fresh weight.

A more pronounced correlation between dark green proportion and fresh weight was observed in the red lettuce experiment than in the green lettuce experiment. This is because red lettuce contains not only chlorophyll but also anthocyanin, and the effect of nitrogen fertilizer is more clearly analyzed than in green lettuce, which is colored only by chlorophyll. In addition, among green RGB colors (i.e., the value of G is higher than the values of R and B), the dark green color may be more distinguishable in red leaves than in green leaves.

The nitrogen content and photosynthetic capacity of leaves are closely related to the duration of light exposure, with light intensity playing an important role (DeJong and Doyle, 1985). The longer the exposure to high light, the greater the nitrogen content of the leaves, resulting in enhanced photosynthetic capacity. Unlike the other three experiments conducted in the spring, the second red lettuce experiment was conducted in the fall season. In the fall, photosynthetic activity was likely limited due to shorter sunlight duration and reduced light intensity. Therefore, the differences in growth among fertilizer treatments were not evident in the fall experiment, possibly due to reduced nitrogen absorption and utilization.

## 5 Conclusion

The objectives of this study were to compare the effects of nitrogen fertilizer, leaf vegetable fertilizer, and microelement fertilizer on the fresh weight and leaf count and to predict the growth responses by leaf color using RGB imaging with ordinary smartphones. The novelty of this work was evaluation of this non-destructive, simple, and economic method for predicting the lettuce yield, and it is the novelty of this work. Based on the four experiments, twice for green-leaf lettuce and twice for red-leaf lettuce, we conclude that the treatment effect is more significant on fresh weight than on leaf count, the nitrogen fertilizer is the most effective for increasing fresh weight, and the fresh weight can be predicted by the dark green proportion extracted from RGB images obtained by commonly used smartphone models.

Predicting fresh weight using RGB imaging enables farmers to adopt precision agriculture more easily, optimizing crop growth while reducing agricultural input costs. Additionally, applying an appropriate amount of fertilizer conserves environments.

RGB imaging can serve as non-destructive tools for rapid assessments of nutritional status of plants, and more reliable cameras and external controls may increase the predictability. In this study, we conclude that the dark green proportion is a more reliable predictor of fresh weight than the normalized intensity value, but we observed substantially different results between smartphone cameras. The measurement reliability should be improved via calibration or adjustment of sensors and studying more various cameras. In addition, more reliable and sensitive color parameters (than the dark green proportion) may be found for better prediction. With continual improvement, this non-destructive, simple, and economic method can lead to user-friendly mobile applications that enable farmers to easily utilize this technology in the field.

## Data Availability

The original contributions presented in the study are included in the article/Supplementary Material. Further inquiries can be directed to the corresponding author.
